# The Iron-chelator, N,N’-bis (2-hydroxybenzyl) Ethylenediamine-N,N’-diacetic acid is an Effective Colistin Adjunct against Clinical Strains of Biofilm-Dwelling *Pseudomonas aeruginosa*

**DOI:** 10.3390/antibiotics9040144

**Published:** 2020-03-27

**Authors:** Karla Mettrick, Karl Hassan, Iain Lamont, David Reid

**Affiliations:** 1 School of Environmental and Life Sciences, University of Newcastle, Callaghan, NSW 2308, Australia; karl.hassan@newcastle.edu.au; 2 Department of Biochemistry, University of Otago, Dunedin 9016, New Zealand; iain.lamont@otago.ac.nz; 3 QIMR-Berghofer Institute of Medical Research, Herston, QLD 4029, Australia; David.Reid@qimrberghofer.edu.au

**Keywords:** iron chelation, biofilm, *Pseudomonas aeruginosa*, HBED, cystic fibrosis

## Abstract

Targeting the iron requirement of *Pseudomonas aeruginosa* may be an effective adjunctive for conventional antibiotic treatment against biofilm-dwelling *P. aeruginosa*. We, therefore, assessed the anti-biofilm activity of N,N’-bis (2-hydroxybenzyl) ethylenediamine-N,N’-diacetic acid (HBED), which is a synthetic hexadentate iron chelator. The effect of HBED was studied using short-term (microtitre plate) and longer-term (flow-cell) biofilm models, under aerobic, anaerobic, and microaerobic (flow-cell) conditions and in combination with the polymyxin antibiotic colistimethate sodium (colistin). HBED was assessed against strains of *P. aeruginosa* from patients with cystic fibrosis and the reference strain PAO1. HBED inhibited growth and biofilm formation of all clinical strains under aerobic and anaerobic conditions, but inhibitory effects against PAO1 were predominantly exerted under anaerobic conditions. PA605, which is a clinical strain with a robust biofilm-forming phenotype, was selected for flow-cell studies. HBED significantly reduced biomass and surface coverage of PA605, and, combined with colistin, HBED significantly enhanced the microcolony killing effects of colistin to result in almost complete removal of the biofilm. HBED combined with colistin is highly effective *in vitro* against biofilms formed by clinical strains of *P. aeruginosa*.

## 1. Introduction

Recalcitrant *Pseudomonas aeruginosa* infection within the lungs of individuals with the genetic disease cystic fibrosis (CF) remains the predominant cause of morbidity and early mortality in this patient population [1]. Iron constitutes a major nutrient for *P. aeruginosa* growth since it is an essential co-factor in multiple core enzyme systems. Iron has also been shown to be a positive regulator of *P. aeruginosa* biofilm formation and is a key controller of virulence factor expression (reviewed by Reference [2]). There are strong suggestions that iron is involved in disease pathogenesis in the CF lung and that the increased iron content of airway secretions promotes *P. aeruginosa* infection [3,4,5].

We have previously demonstrated that targeting *P. aeruginosa* iron acquisition with biological and synthetic iron chelators inhibits biofilm formation, particularly under anaerobic conditions [6,7]. *P. aeruginosa* employs multiple mechanisms of iron acquisition within the CF lung, which further highlights the importance of iron to this pathogen in the disease state. However, this also offers the opportunity to intervene therapeutically [8,9,10]. 

In this paper, we assess the anti-biofilm effects of the hexadentate synthetic ferric iron chelator, N,N’-bis (2-hydroxybenzyl) ethylenediamine-N,N’-diacetic acid (HBED), on laboratory and clinical strains of *P. aeruginosa*. HBED, an iron chelator first described more than 50 years ago [11], has a high affinity (10^40^ M^-1^) and selectivity for ferric iron but, despite its low toxicity [12], the zwitterionic nature of HBED results in low bioavailability that has, thus far, prevented its clinical use as a systemic drug [13].The potential for HBED to sequester Fe^3+^ with an affinity that *P. aeruginosa* cannot overcome is anticipated to cause iron starvation and effects such as growth inhibition. We were particularly interested in the ability of HBED to disrupt mature, established *P. aeruginosa* biofilms and examined the efficacy of HBED against biofilm-dwelling bacteria when combined with the conventional polymyxin antibiotic colistin.

## 2. Results

### 2.1. HBED Decreases Aerobic and Anaerobic Biofilm Formation of P. aeruginosa 

**Figure 1:**
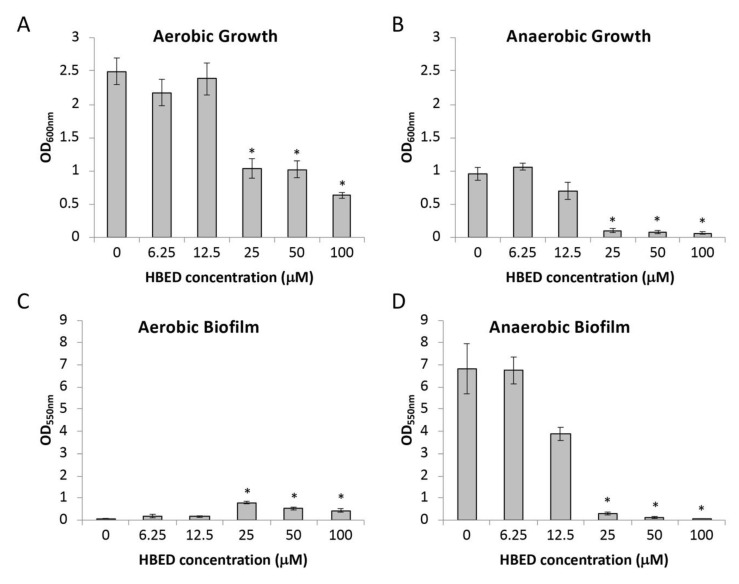
Effect of N,N’-bis (2-hydroxybenzyl) ethylenediamine-N,N’-diacetic acid (HBED) on strain PAO1 (**A**,**B**) growth levels, and (**C**,**D**) short-term biofilm formation. Growth was under aerobic (**A**,**C**) and anaerobic conditions (**B**,**D**) in succinate media with 10 μM ferric chloride. Values shown represent the mean level of growth or biofilm formation +/− standard error of the mean (SEM). * denotes significantly different values in the presence of HBED than without the chelator (95% confidence) determined using analysis of variance (ANOVA) with Dunnett’s Method post hoc test.

We first investigated whether HBED could impair growth and biofilm development of *P. aeruginosa* strain PAO1 in a microtiter plate assay (Figure 1). Under aerobic conditions in a minimal medium (succinate medium supplemented with 10 μM FeCl_3_), HBED had inhibitory effects on the growth of planktonic cells at levels of 25 μM and above (Figure 1A). The biofilm level at these concentrations was increased compared to the absence of chelator (Figure 1C). Growth of PAO1 was impaired by HBED at the same concentrations under anaerobic conditions (Figure 1B) as in aerobic conditions (Figure 1A). Basal levels of biofilm formation were much higher under anaerobic conditions compared to the presence of oxygen. However, HBED greatly impaired biofilm formation, which is consistent with growth inhibition (Figure 1D).

### 2.2. Inhibition of Growth and Biofilm Formation by HBED is Iron-Dependent

**Figure 2:**
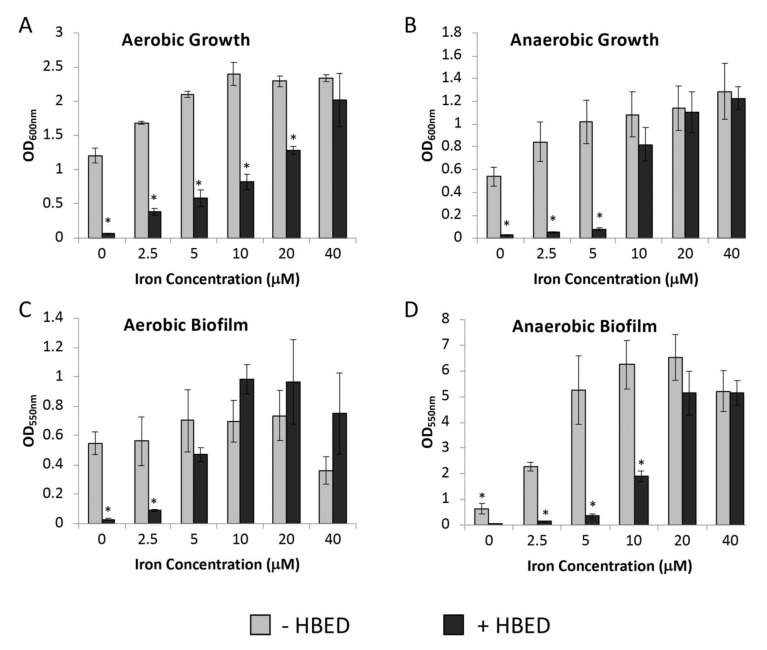
Effect of supplemental iron on growth and biofilm formation of PAO1 in the presence of N,N’-bis (2-hydroxybenzyl) ethylenediamine-N,N’-diacetic acid (HBED). Values shown represent the mean level of (**A**,**B**) growth and (**C**,**D**) biofilm formation +/− standard error of the mean (SEM) of the *P. aeruginosa* strain PAO1 in succinate medium with FeCl_3_ supplemented at concentrations indicated in the presence and absence of HBED (25 µM in aerobic conditions, 10 µM in anaerobic conditions). Growth was under aerobic (a, c) and anaerobic (b, d) conditions. * denotes significantly different values compared to no HBED determined using Student’s *t*-test (*P* < 0.05).

To confirm that HBED was acting in an iron-dependent manner to decrease growth and biofilm formation, bacterial growth was measured in succinate medium containing a set concentration of HBED and supplemented with increasing concentrations of iron (Figure 2). Addition of iron overcame the effectiveness of HBED in inhibiting the growth of strain PAO1 under both aerobic and anaerobic culture conditions (Figure 4A,B) with 40 µM of FeCl_3_ required to fully reduce the effects of 25 µM HBED in the presence of oxygen and 10 µM of FeCl_3_ being sufficient to reduce the inhibitory effects of 10 µM HBED under anaerobic conditions. HBED binds Fe^3+^ with a stoichiometry of 1:1 and, therefore, these concentrations are what is reasonably expected to overcome the amount of HBED present. A lower concentration of HBED (10 μM) was used in anaerobic conditions to reflect the increased impairment of growth and biofilm by the chelator in the absence of oxygen (Figure 1). Supplementary iron also reversed the effects of HBED on biofilm formation under both aerobic and anaerobic conditions (Figure 2C,D). Additionally, 40 µM of iron caused a decrease in biofilm formation under aerobic conditions (Figure 2C). This is consistent with previous findings that iron can inhibit biofilm formation [14] with our observations that HBED increased biofilm in the presence of oxygen (Figure 1C).

**Figure 3:**
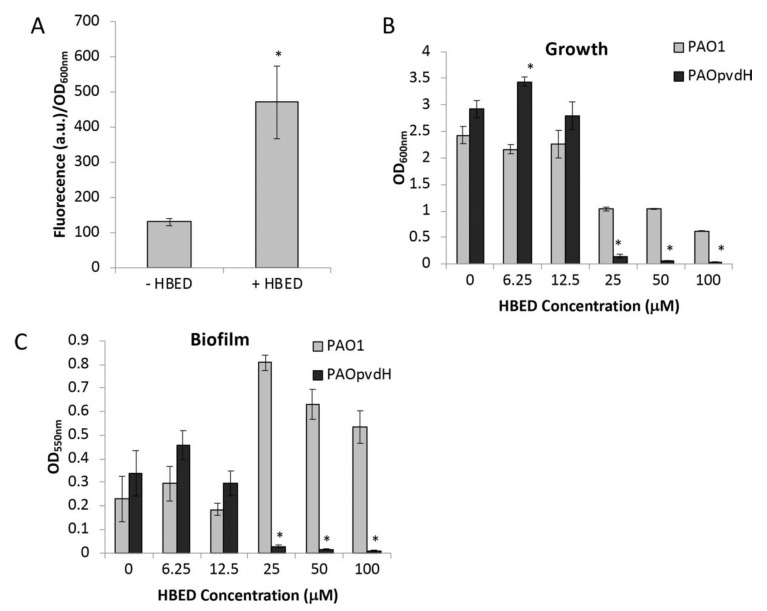
Effect of N,N’-bis (2-hydroxybenzyl) ethylenediamine-N,N’-diacetic acid (HBED) on pyoverdine production in PAO1. (**A**) Pyoverdine production from PAO1 grown in succinate medium with 10 μM FeCl_3_ in the presence and absence of 25 µM HBED. The values represent the mean levels of fluorescence (405 nm/460 nm) adjusted for growth (OD_600 nm_) +/− standard error of the mean (SEM) of three biological replicates. (**B**) Growth and (**C**) biofilm formation of PAO1 and the pyoverdine deficient mutant PAO*pvdH* in succinate medium with 10 μM FeCl_3_ with HBED at concentrations indicated. * denotes significantly different values compared to PAO1 with the equivalent concentration of HBED determined using Student’s *t*-test (*P* < 0.05).

*P. aeruginosa* exports a fluorescent siderophore, pyoverdine, in response to iron deprivation. Fluorescence can be used as a measure of pyoverdine production and, thus, be used as an indicator for iron deprivation. Both the stability constant of HBED for ferric iron (K_a_ = 10^40^ M^-1^) [11] and its pFe value (31) [15] are considerably higher than those of pyoverdine (K_a_ = 10^32^ M^-1^, pFe = 27) [16,17], which suggests that pyoverdine would not be able to effectively compete with HBED to acquire iron that had been chelated by HBED. We determined whether iron starvation induced by HBED would stimulate pyoverdine production (Figure 3A). As anticipated, the presence of 10 μΜ HBED was sufficient to cause an increase in pyoverdine-dependent fluorescence. To investigate the role of pyoverdine in overcoming an exogenous iron-chelator, a pyoverdine deficient mutant (PAO*pvdH*) was grown in the presence and absence of HBED. Under aerobic conditions, the results show HBED decreased aerobic growth of the pyoverdine mutant to a greater degree than the parental PAO1 strain (Figure 3B). Furthermore, biofilm formation by the pyoverdine mutant was significantly decreased in the presence of HBED at concentrations greater of 12.5 µM compared to the wild type (Figure 3C). 

These results, therefore, indicate that, in minimal media, HBED deprives *P. aeruginosa* of iron, which results in decreased growth, increased pyoverdine production and, under anaerobic conditions, decreased biofilm formation. The presence of pyoverdine is key for the susceptibility of *P. aeruginosa* to HBED-induced iron starvation.

### 2.3. HBED Decreases Growth of P. aeruginosa in Sub-inhibitory Concentrations of Antibiotics

To investigate whether the presence of HBED decreases the minimal inhibitory concentration (MIC) of conventional antibiotics, MIC microdilution assays were undertaken in Mueller Hinton Broth (MHB) in the presence of HBED. MHB was used in these assays because it is the standard media used in MIC testing, as recommended by the Clinical and Laboratory Standards Institute (CLSI) and European Committee on Antimicrobial Susceptibility Testing (EUCAST) [18]. The concentration of iron in MHB is approximately 10 μM [19]. To ensure an appropriate concentration of HBED was added to this media, growth and biofilm formation analysis of PAO1 in MHB with HBED was undertaken (Figure S1). Similar to minimal media, HBED was able to significantly decrease anaerobic growth and biofilm formation of PAO1 (Figure S1B,D). No significant decrease in growth was seen under aerobic conditions but a small decrease in biofilm formation was seen with 1 mM HBED (Figure S1B,D). Despite no decrease in aerobic growth, pyoverdine production was increased in the presence of HBED at concentrations as low as 4 μM and growth and biofilm formation of the pyoverdine deficient mutant was significantly reduced (Figure S2). These results indicate HBED causes iron deficiency in this media and, subsequently, concentrations of 25 μM and 10 μM HBED were selected for the MIC assay under aerobic and anaerobic conditions, respectively. 

Colistin, ciprofloxacin, and gentamicin were used as representative members from the polymyxins, fluoroquinolones, and aminoglycosides antimicrobial classes, which have distinct modes of action and growth in the presence of these with HEBD under aerobic and anaerobic conditions were assessed (Table 1).

Under aerobic conditions, no decrease in MIC was seen for any of the antibiotics when 25 μM HBED was added into the media. However, at antibiotic concentrations that are 2-fold to 4-fold below MIC, aerobic growth of PAO1 in the presence of HBED decreased considerably compared to the control (Figure S3). In the absence of oxygen, the presence of 10 μM chelator decreased the MIC of both colistin and gentamicin two-fold (Table 1). Consistent with the inhibitory effect of HBED on anaerobic growth in MHB (Figure S1B), growth of PAO1 was reduced across all sub-inhibitory levels of antibiotics compared to the control, but, as with growth in aerobic conditions, growth was decreased further at concentrations 2-fold to 4-fold below the MIC (Figure S3). Taken together, these results suggest HBED could increase the clearance of *P. aeruginosa* at sub-inhibitory concentrations of antibiotics.

### 2.4. HBED Decreases Growth and Biofilm Formation of P. aeruginosa Clinical Isolates

Although strain PAO1 is a model bacterium in biofilm and CF research, it possesses many different characteristics to current clinical *P. aeruginosa* isolates obtained from CF patients [20,21,22]. *P. aeruginosa* undergo genetic and phenotypic adaptations during chronic infection of the CF lung, which result in unique characteristics that need to be considered as new therapeutics are developed for use in the clinical setting [23,24,25,26]. We, therefore, studied isolates of *P. aeruginosa* from CF patients. This includes Australian epidemic strains AUST01, AUST02, and AUST03 and strain PA605 (Table 2). PA605 is a "first isolate" obtained from a child as part of an epidemiological study of *P. aeruginosa* in the Tasmanian CF population. We also assessed “early” and “late” isolates where the “early” isolates were obtained from children and the “late” isolates were obtained from CF adults with long-term infection (Table 2). The early and late epidemic strains were collected from different patients and not from the same patient over time.

**Figure 4:**
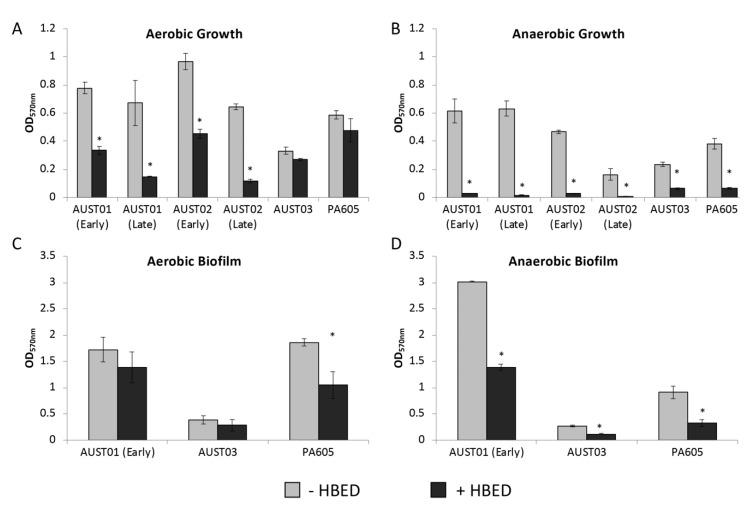
Effect of N,N’-bis (2-hydroxybenzyl) ethylenediamine-N,N’-diacetic acid (HBED) on growth and biofilm formation of cystic fibrosis (CF) isolates. Values shown represent the mean level of (**A**,**B**) growth and (**C**,**D**) biofilm formation +/− standard error of the mean (SEM) of the *P. aeruginosa* strains AUST01, AUST02, AUST03, and PA605 in succinate medium with 10 µM FeCl_3_ in the presence and absence of 100 µM HBED. Growth was under aerobic (**A**,**C**) and anaerobic (**B**,**D**) conditions. * denotes significantly different values compared to untreated values determined using Student’s *t*-test (*P* < 0.05).

Overall, the growth of clinical CF strains in succinate medium was significantly impaired by HBED with inhibition of aerobic and anaerobic planktonic growth ranging from 19–97% (Figure 4). As observed with PAO1 (Figure 1), the magnitude of these inhibitory effects was greater under anaerobic conditions (74–97%) (Figure 4A) compared to aerobic conditions (19–82%) (Figure 4B). The greatest inhibition of aerobic growth was seen with strains AUST01 and AUST02 and was most evident with the late strains (Figure 4). 

Except for AUST01 (early), AUST03, and PA605, the clinical CF isolates did not form robust biofilms in 96-well microtitre plates (data not shown). In contrast to findings with strain PAO1, where aerobic biofilm was enhanced and anaerobic biofilm decreased (Figure 1C,D), HBED caused a decrease in biofilm formation for AUST01 (early), AUST03, and PA605 under both aerobic and anaerobic growth conditions (Figure 4C,D). 

### 2.5. HBED is Effective against Mature Biofilms and Increases the Efficacy of Colistin

The above experiments indicated that HBED is effective against short-term biofilms, but, during chronic lung infection with *P. aeruginosa* in CF, biofilm aggregates are long-lasting and recalcitrant to antibiotic therapy [29]. Mature biofilms are comprised of complex communities of bacteria that behave very differently to planktonic cells [30]. Established biofilms are not only resistant to therapy, but likely also provide the source of recrudescence of infection after conventional antibiotic therapy [31,32]. To model mature biofilms, we used a flow-cell biofilm system [33]. CF strain PA605 was chosen for these experiments since it formed robust biofilms *in vitro* in the short-term 96-well plate screening experiments that were decreased in the presence of HBED under both aerobic and anaerobic conditions (Figure 4). As well as investigating the effectiveness of HBED alone in disrupting mature biofilms, we investigated the effect of HBED in combination with colistin, which is a polymixin antibiotic that is commonly used as an inhaled treatment for *P. aeruginosa* respiratory infections. Colistin has been shown to be effective against anaerobically grown *P. aeruginosa* and bacterial cells located within the depths of mature biofilms [34,35]. Furthermore, the MIC of colistin was decreased under anaerobic conditions in the presence of HBED (Table 1). The concentration of colistin selected (10 mg/L) was within the range achievable within the lung therapeutically, but below the concentration required for killing colistin-susceptible strains of *P. aeruginosa in vitro* [36,37].

**Figure 5:**
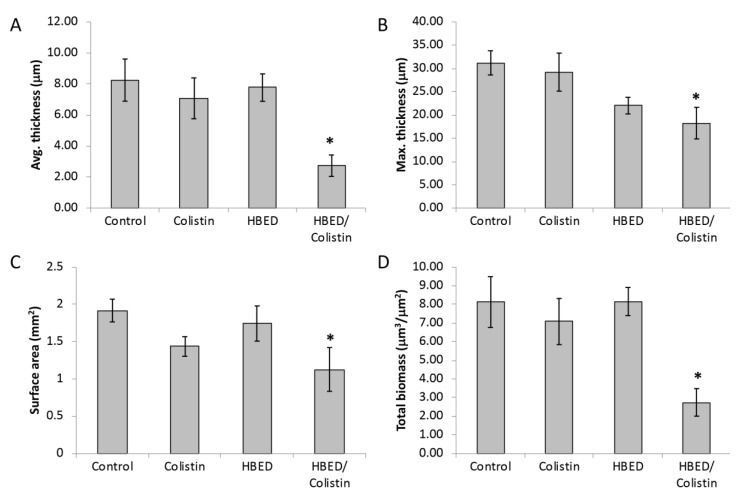
COMSTAT analysis of *P. aeruginosa* biofilms. *P. aeruginosa* PA605 was grown for a total of 10 days with treatment (HBED (100 µM) and/or colistin (10 mg/L)) being incorporated into the media after six days. Biofilms were stained with LIVE/DEAD^®^ stain and viewed using confocal scanning laser microscopy (CSLM). (**A**) Average biofilm thickness. (**B**) Maximum biofilm thickness. (**C**) Surface area of biomass. (**D**) Total biomass. *Significantly different from the control (95% confidence) determined using ANOVA with Dunnett’s Method post hoc test. The values are the means +/− standard error of the mean (SEM) of at least three independent experiments.

*P. aeruginosa* PA605 biofilms were allowed to form and grow for six days in a flow-cell at which point HBED and/or colistin was added and treatment continued for a further four days. Neither HBED nor colistin on their own significantly decreased average thickness, maximum thickness, surface area, or total biomass of mature biofilms (Figure 5). In combination, HBED and colistin decreased the thickness (Figure 5A,B) and surface area (Figure 5C) of the biofilm. The most striking result was seen when HBED and colistin were delivered in combination, which caused a large decrease in total biomass of the biofilm (Figure 5D). A similar decrease in thickness and biomass was seen for biofilms that were grown for a total of three days with HBED and antibiotic treatment was incorporated into the growth media in the final 24 h (Figure S4). 

The impact of the combined treatment after 10 days can be seen visually in Figure S4. Following treatment, only a few adherent bacterial cells remained with large areas of exposed flow-cell surface visible and minimal residual biofilm matrix. Furthermore, this disruption of the biofilm was accompanied by an increased proportion of dead cells, as determined by the LIVE/DEAD^®^ stain, which was not seen when the two interventions were administered individually (Figure S5).

## 3. Discussion

Chronic infection with biofilm-forming antibiotic resistant *P. aeruginosa* presents a major treatment challenge in individuals with CF. The importance of iron to *P. aeruginosa* and the emerging data that demonstrate iron homeostasis is abnormal in the CF lung suggests that therapeutics targeting bacterial iron uptake pathways may be particularly useful adjuncts to existing conventional antibiotics in this disease. This study demonstrates that, in minimal media, the hexadentate synthetic iron chelator, N,N’-bis (2-hydroxybenzyl) ethylenediamine-N,N’-diacetic acid (HBED), inhibits biofilm formation by *P. aeruginosa* isolates obtained from patients with CF, including highly prevalent epidemic strains. The most marked effects were observed when HBED was combined with colistin in a microaerobic biofilm flow-cell model with the two compounds acting synergistically to cause almost complete disruption of mature biofilms accompanied by substantial killing of resident colonies (Figure 5, Figure S5). 

The low bioavailability of HBED [13] would be highly advantageous for an inhaled iron chelator designed to exclusively target the respiratory tract. Ferric iron chelators targeting *P. aeruginosa* biofilms have been investigated previously [6,38,39,40]. However, a ferrous iron chelator or a Fe^2+^/Fe^3+^ chelator may be safer and more effective [2,41,42]. While HBED has been shown to have high affinity for Fe^3+^, the association constant with Fe^2+^ has been difficult to determine because HBED rapidly oxidises it to Fe^3+^, even under anaerobic conditions [11,43]. The potential to chelate Fe^2+^ may explain why HBED was so effective in this study under anaerobic and microaerophilic conditions.

Several factors need to be considered when assessing the efficacy of iron-targeting interventions in the CF lung. First, oxygen tensions within lung niches in established bronchiectasis may range between aerobic, microaerobic, and even anaerobic in mucous occluded airways [44]. We, therefore, assessed HBED under aerobic, microaerobic, and anaerobic conditions using short-term growth and short-term and long-term biofilm assays. The iron-chelating mode of action for HBED was confirmed by the demonstration that supplemental iron abolished HBED’s growth inhibitory effects and also ameliorated the increased synthesis of pyoverdine by strain PAO1 in the presence of HBED. The exclusive specificity of HBED for iron in succinate medium was confirmed by LCMS (data not shown). Under aerobic conditions, growth inhibition by HBED was variable between strains (Figure 1 and Figure 4). The HBED-induced increase in pyoverdine production by *P. aeruginosa* (Figure 3A) combined with the decreased fitness of a pyoverdine null mutant in the presence of HBED (Figure 3B,C) suggests that this siderophore may enable *P. aeruginosa* to overcome iron starvation imposed by synthetic iron chelators. Different strains of *P. aeruginosa* have different capacities to produce pyoverdine as well as other iron-acquisition systems, which may explain the strain-specific effects of HBED on growth that we observed. AUST03 and PA605 had both the smallest HBED-induced decrease in aerobic growth (Figure 4A), which suggests they may have been less iron-starved by the presence of HBED. Other systems within *P. aeruginosa* may decrease the bacteria’s reliance on pyoverdine for iron uptake. Phenazines are known to reduce Fe^3+^ and the presence of ferrous iron has been correlated with phenazine production [41]. 

Importantly, strains of *P. aeruginosa* from CF patients were susceptible to growth inhibition by HBED under both aerobic and anaerobic conditions. HBED was effective against important epidemic strains of *P. aeruginosa* that are highly prevalent in Australia. HBED was also effective at inhibiting growth and biofilm formation of strain PA605, which is a non-epidemic strain obtained from a young CF patient. PA605 was of particular interest given that the early stages of infection with *P. aeruginosa* in the CF lung may be the only opportunity for anti-microbial interventions to eradicate *P. aeruginosa* and prevent chronic infection [45]. Inhibition of growth and biofilm formation for all strains tested was most marked under anaerobic conditions. These findings may have important clinical relevance as several conventional antibiotics used in CF by the inhalation route such as tobramycin, amikacin, and aztreonam are less effective under reduced oxygen conditions [46]. Antibiotic adjuncts such as iron chelators that are effective in diverse oxygen environments may, therefore, substantially enhance the bactericidal effects of conventional antimicrobials. The increased susceptibility of *P. aeruginosa* to HBED under anaerobic conditions may be explained by the fact that its main ferric iron acquisition pathway (pyoverdine) is inactive under anaerobic conditions [47], where a substantial proportion of the iron present will be in the reduced ferrous form. Recent data suggest that *P. aeruginosa* employs ferrous iron uptake mechanisms in the CF lung through FeoB, but the mechanism has not been fully characterised [8,41]. However, why HBED was so effective under anaerobic conditions, where more ferrous iron would be theoretically available to *P. aeruginosa*, is not clear, but may be due to chelation and oxidation of ferrous iron.

A potential downside of therapeutically imposing iron starvation during treatment is stimulation of *P. aeruginosa* virulence [9]. In particular, sigma factor PvdS is strongly iron-regulated and conditions of iron depletion would be expected to cause up-regulation of PvdS and increased production of the PvdS-regulated virulence factors pyoverdine, exotoxin A, and the endoprotease PrpL [48]. However, the results suggesting HBED could not outcompete pyoverdine in aerobic conditions (Figure 3), but was highly effective in anaerobic conditions, suggests that pyoverdine and the associated virulence pathway was not up-regulated under these conditions. It is possible that any bacteria dispersed from the biofilm following HBED treatment will demonstrate up-regulated virulence will be either rapidly targeted by the host immune system or susceptible to antibiotic action given the loss of biofilm protection. However, this requires further investigation in animal models of CF. Furthermore, we used sub-inhibitory concentrations of colistin in the flow cells as a proof of concept, but higher concentrations of antibiotic delivered by inhalation may be achieved within the CF airway, which will further facilitate enhanced killing of cells as they are dispersed from biofilms. 

In summary, our findings suggest HBED as a combination therapy with colistin is a potential anti-biofilm strategy. 

## 4. Materials and Methods 

### 4.1. Bacterial Strains 

*P. aeruginosa* laboratory strain PAO1, originally isolated from a burn wound [49], and the PAO*pvdH* mutant [50] were from laboratory stocks. Clinical strains used in this study were isolated from adult CF individuals with chronic *P. aeruginosa* infection [27,28] (Table 2). *P. aeruginosa* strain PA605 was obtained at the time of first isolation of *P. aeruginosa* from the sputum of a child with CF and was provided by Louise Roddam (University of Tasmania, Australia). Australian Epidemic Strains, AUST01 (“Melbourne clone”), and AUST02 (“Brisbane clone”) were isolated from sputum or bronchoalveolar lavage samples of CF patients as part of an Australian national epidemiological study (Table 2) [27]. Epidemic strains were chosen because of their widespread distribution in the pediatric and adult CF populations in Australia and their association with increased treatment requirements and antibiotic resistance.

### 4.2. Growth Media

Strains were grown from storage on Nutrient Agar for 16-18 h under aerobic conditions. Bacterial growth and biofilm assays in microtiter plates were then conducted in BBL^TM^ Mueller Hinton Broth, BD (MHB) or a minimal succinate medium (K_2_HPO_4_ 6 g/L, KH_2_PO_4_ 3 g/L, (NH_4_)_2_SO_4_ 1 g/L, MgSO_4_.7H_2_O 0.2 g/L, Succinic acid 4 g/L, and adjusted to pH 7.0 with NaOH) [16] as indicated. MHB and succinate media were supplemented with 1% KNO_3_ for growth under anaerobic conditions. Iron (FeCl_3_) was supplemented to succinate medium from stock solutions at 10 µM unless stated otherwise. Anaerobic incubation was undertaken using Biomérieux GENbag anaerobic sachets or AnaeroGen^TM^ compacts (Oxoid). Continuous media flow biofilm assays were conducted in M63 medium (adapted from Reference [51]) (15 mM (NH_4_)_2_SO_4_, 22 mM KH_2_PO_4_, 40 mM K_2_HPO_4_, 5.5 mM glucose, 1 mM MgSO_4_) supplemented with 10 µM iron to supply bacteria with an iron concentration similar to that found within the CF lung [3,52]. N,N’-bis (2-hydroxybenzyl) ethylenediamine-N,N’-diacetic acid (HBED) was purchased from Strem Chemicals. Colistin sulfate and ciprofloxacin were purchased from Sigma. Gentamicin sulfate was purchased from AG Scientific.

### 4.3. Biofilm Assays

#### 4.3.1. Short-Term Biofilm Assays of PAO1

Biofilm formation was examined in a modified microtiter plate assay using crystal violet as described previously [53]. Overnight *P. aeruginosa* cultures grown in LB were diluted to OD_600nm_ = 0.001 in the desired medium with appropriate supplements. A total of 100 µL aliquots were dispensed into 96-well polystyrene microtiter plates (Greiner). A minimum of four technical replicates within each experiment was carried out and each experiment was reproduced at least three times. Bacteria were incubated (37 °C) under aerobic and anaerobic conditions for 20 h. Total bacterial growth was determined using optical density (600 nm) in a PHERAstar FS plate reader (BMG Labtech). Biofilms were washed and stained with 0.1% (w/v) crystal violet (CV) for 15 min at room temperature. After vigorous washing with water, the stained biofilms were made soluble with 30% acetic acid and the biofilm density determined by absorbance (550 nm). 

#### 4.3.2. Short-term Biofilm Assays of Clinical Isolates

Biofilm formation of the *Pseudomonas aeruginosa* clinical isolates was as described in 4.3.1 with the following modifications. Overnight cultures were diluted to OD_600 nm_ = 0.01 in succinate medium with 10 μM FeCl_3_. 200 μL aliquots were dispensed into 96-well polystyrene microtiter plates (Greiner). Total bacterial growth was determined using optical density (570 nm) in a microplate reader (Molecular Devices SpectraMax^®^ M2). After biofilms were stained and washed, biofilms were made soluble with 100% ethanol and the biofilm density of 200 µL aliquots was determined by absorbance (570 nm). Anaerobic incubation was undertaken in anaerobe bags using AnaeroGenTM compacts (Oxoid).

#### 4.3.3. Flow Cell Biofilm Studies

For the continuous flow model, biofilms were cultivated at 37 °C in three channel flow-cells with individual channel dimensions of 0.3 × 4 × 40 mm, as described elsewhere [32,54]. For inoculation of bacteria, medium flow was arrested and the inoculum (0.5 ml of overnight grown broth culture diluted to OD_600nm_ = 1) was introduced into the flow-cell. After inoculation, flow-cells were inverted (2 h) to allow bacterial adherence. The flow cell was then returned upright and media flow was started at 100 µL/min (1 h) to wash through any non-adherent bacteria. Flow was then maintained at a velocity of 50 µL/min. Bacteria were grown for six days at which point colistin (10 mg/L) and/or HBED (100 µM) were introduced into the flow media and bacterial growth was allowed to continue for a further four days. 

At the conclusion of long-term assays, the biofilms were stained using SYTO^®^9 (Invitrogen) and examined by confocal scanning laser microscopy (CSLM, Zeiss LSM 510 and its Zen operating software) using an argon 488-nm laser and a 20×/1.0 DIC VIS IR W plan-apochromat (Zeiss) water immersion objective. Representative images were analysed using the COMSTAT software [55]. For determining viability, biofilms were stained with the LIVE/DEAD^®^ BacLight™ Bacterial Viability Kit (Invitrogen) and images were captured using a Leica DM LB2 fluorescence microscope with a cooled CCD Magnafire (Optronics) camera.

### 4.4. Pyoverdine Measurement

To determine the concentration of pyoverdine in planktonic cultures, cells were collected by centrifugation, passed through a 0.2 μm filter, and the relative pyoverdine concentration present in the filtered supernatant determined by measuring the fluorescence, as described previously [56]. Fluorescence was detected on a Cary eclipse fluorometer using an excitation wavelength of 405 nm (excitation slit width = 5) and a scanning emission of 425 m to 530 nm (emission slit width = 10). PMT was 600. For simplicity, only emission at 460 nm is presented in this case, which has been adjusted for relative bacterial density using absorbance (600 nm).

### 4.5. Minimal Inhibitory Concentration

Minimal inhibitory concentrations of colistin sulfate, gentamicin sulfate, and ciprofloxacin were determined using broth microdilution, as described by Reference [18]. Assays were carried out in MHB media supplemented with 20 mg/L Ca^2+^ and 10 mg/L Mg^2+^ (cation adjusted). HBED was added at a final concentration of 25 μM under aerobic conditions and 10 μM for anaerobic conditions. To avoid the adsorption of colistin sulfate to the microtiter plate, polysorbate 80 was added at a final concentration of 0.002% (v/v). Microtiter plates incubated in aerobic conditions were sealed with a Breathe-Easy^®^ membrane (Diversified Biotech) and grown with shaking. Total bacterial growth was determined using optical density (600 nm) in a PHERAstar FS plate reader (BMG Labtech).

### 4.6. Statistical Analysis

Data shown represent the mean ± SEM of at least three biological replicates. The significance of the results was determined using either the Student’s t test or one-way analysis of variance (ANOVA) with the Dunnett method and 95% confidence post hoc test (Minitab^®^ 19). *P-*values below 0.05 were considered significant.

## Figures and Tables

**Table 1:** Minimal inhibitory concentrations of antibiotics in the presence of N,N’-bis (2-hydroxybenzyl) ethylenediamine-N,N’-diacetic acid (HBED).

Antibiotic	Aerobic − HBED	Aerobic + 25 μM HBED	Anaerobic − HBED	Anaerobic + 10 μM HBED
Colistin (mg/L)	0.5	0.5	2	1
Ciprofloxacin (mg/L)	0.25	0.25	0.5	0.5
Gentamicin (mg/L)	2	2	8	4

**Table 2:** Properties of *P. aeruginosa* clinical isolate strains used in this study.

Strain	Isolate Type^1^	Patient Location	Patient Age at Time of Isolation	Reference
PA605	First Isolate	Hobart, TAS	7	This study
AUST01	Early Isolate	Melbourne, VIC	10	[27]
AUST01	Late Isolate	Perth, WA	39	[27]
AUST02	Early Isolate	Brisbane, QLD	2	[27]
AUST02	Late Isolate	Perth, WA	28	[27]
AUST03	Late Isolate	Hobart, TAS	26	[28]

^1^ First isolate – isolate from first detection of *P. aeruginosa* infection. Early isolate – obtained from a child with cystic fibrosis (CF). Late isolate – obtained from an adult with CF.

## Supplementary Materials

The following are available online at https://www.mdpi.com/2079-6382/9/4/144/s1. Figure S1: Effect of N,N’-bis (2-hydroxybenzyl) ethylenediamine-N,N’-diacetic acid (HBED) on strain PAO1 growth levels, and short-term biofilm formation. Figure S2: Effect of N,N’-bis (2-hydroxybenzyl) ethylenediamine-N,N’-diacetic acid (HBED) on pyoverdine production in PAO1. Figure S3: Minimal inhibitory concentration analysis of *P. aeruginosa*. Figure S4: COMSTAT analysis of *P. aeruginosa* biofilms. Figure S5: Effect of colistin and N,N’-bis (2-hydroxybenzyl) ethylenediamine-N,N’-diacetic acid (HBED) on established *P. aeruginosa* PA605 biofilms.
