# Supplementary material for: The Iron-chelator, N,N’-bis (2-hydroxybenzyl) Ethylenediamine-N,N’-diacetic acid is an Effective Colistin Adjunct against Clinical Strains of Biofilm-Dwelling Pseudomonas aeruginosa
Source: Antibiotics (Basel). 2020 Mar 27;9(4):144. doi: 10.3390/antibiotics9040144 (PMC7235823; doi:10.3390/antibiotics9040144)
Supplement: Supplementary file 1 [file antibiotics-09-00144-s001.pdf]

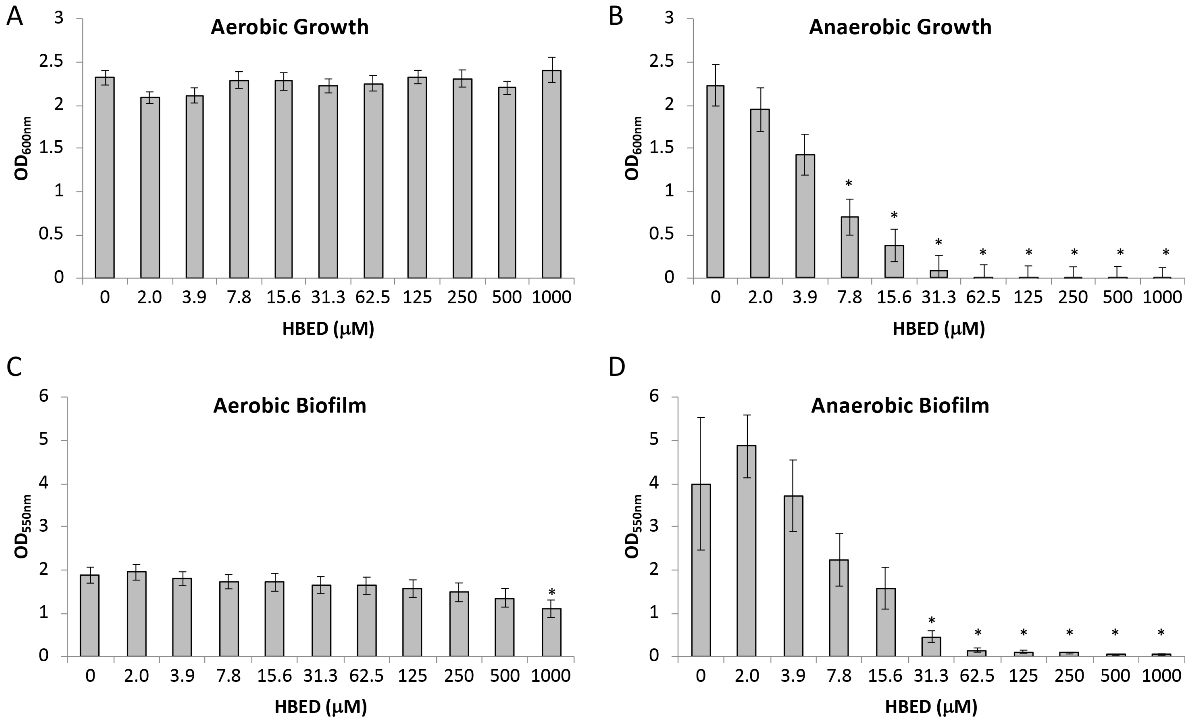


**Figure S1. Effect of N,N′-bis (2-hydroxybenzyl) ethylenediamine-N,N′-diacetic acid (HBED) on strain PAO1 (A, B) growth levels, and (C, D) short-term biofilm formation.** Growth was under aerobic (A, C) and anaerobic conditions (B, D) in Mueller Hinton Broth (MHB) media for 24 hours. Values shown represent the mean level of growth or biofilm formation +/- standard error of the mean (SEM) of at least three biological replicates. * denotes significantly difference values in the presence of HBED than without the chelator (95% confidence) determined using ANOVA with Dunnett's Method post hoc test.

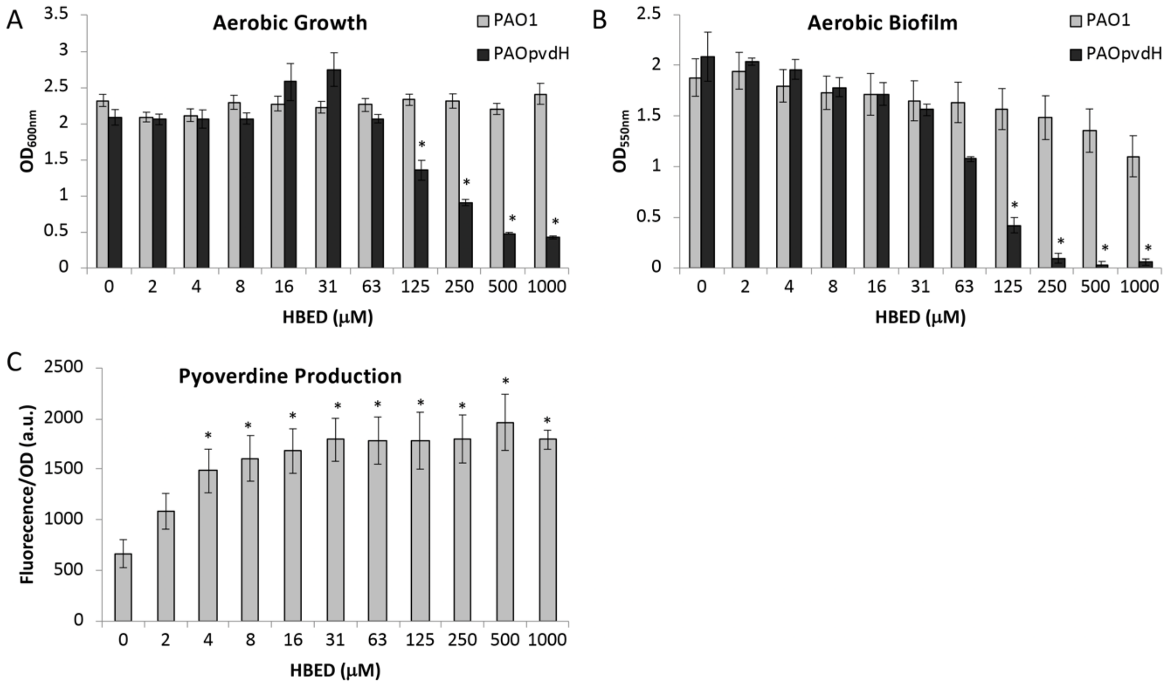


**Figure S2. Effect of N,N′-bis (2-hydroxybenzyl) ethylenediamine-N,N′-diacetic acid (HBED) on pyoverdine production in PAO1.** (A) Aerobic growth and (B) Biofilm formation of PAO1 and the pyoverdine deficient mutant PAO*pvdH* in Mueller Hinton Broth (MHB) with HBED at concentrations indicated. * denotes significantly different values compared to PAO1 with the equivalent concentration of HBED ($P < 0.05$). (C) Pyoverdine production from PAO1 grown in MHB in the presence of HBED as indicated. The values represent the mean levels of fluorescence (405nm/460nm) adjusted for growth ($OD_{600nm}$) +/- standard error of the mean (SEM) of three biological replicates. * denotes significantly different values compared to 0 μM HBED ($P < 0.05$) determined using Student's t test.

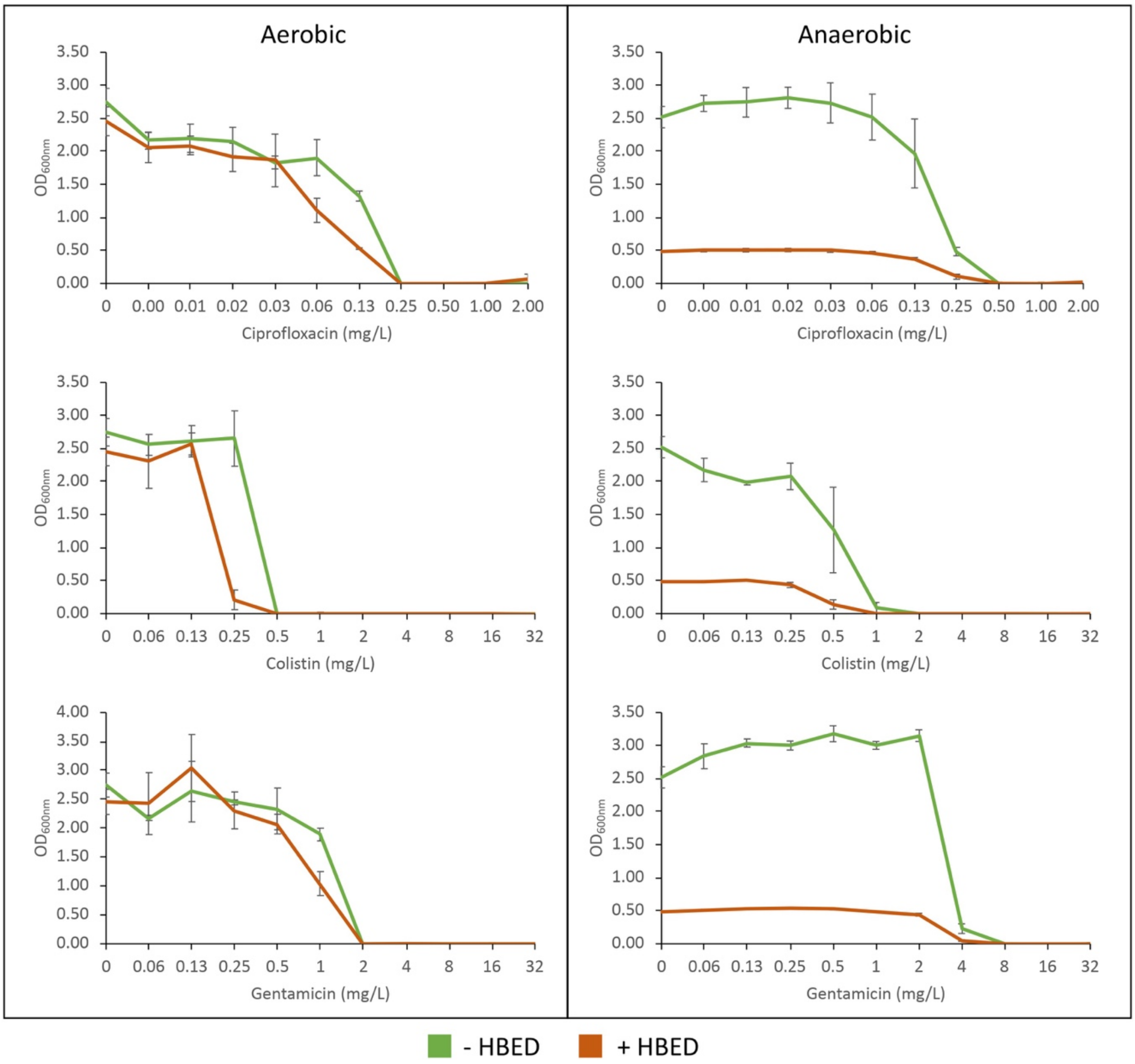


**Figure S3. Minimal inhibitory concentration analysis of *P. aeruginosa*.** PAO1 was grown in cation-adjusted Mueller Hinton Broth (MHB) in aerobic (left panel) and anaerobic (right panel) conditions. Ciprofloxacin, colistin or gentamicin were added at the concentrations as indicated. HBED was added at 25 μM (aerobic) or 10 μM (anaerobic).

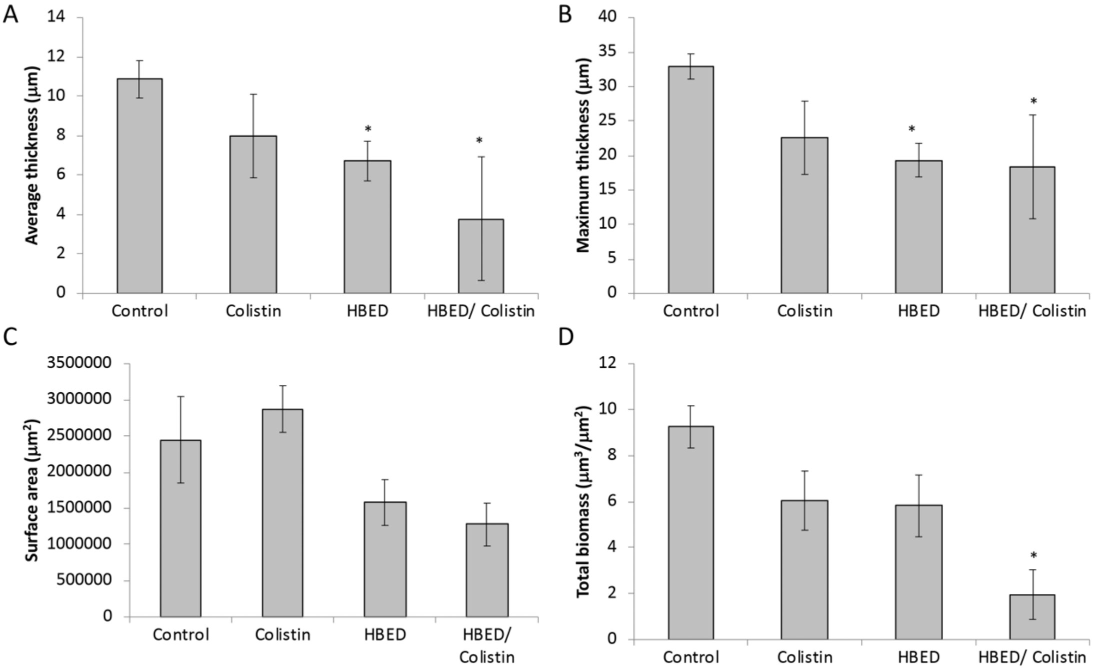


**Figure S4. COMSTAT analysis of *P. aeruginosa* biofilms.** *P. aeruginosa* PA605 was grown for a total of 3 days with treatment (HBED (100 µM) and/or colistin (10 mg/L)) being incorporated into the media after 2 days. Biofilms were stained with LIVE/DEAD® stain and viewed using confocal scanning laser microscopy (CSLM). (A) Average biofilm thickness (B) Maximum biofilm thickness (C) Surface area of biomass (D) Total biomass. *Significantly different from control ($p<0.05$) determined using Student's t test. The values are the means (SEM) of at least two independent experiments.

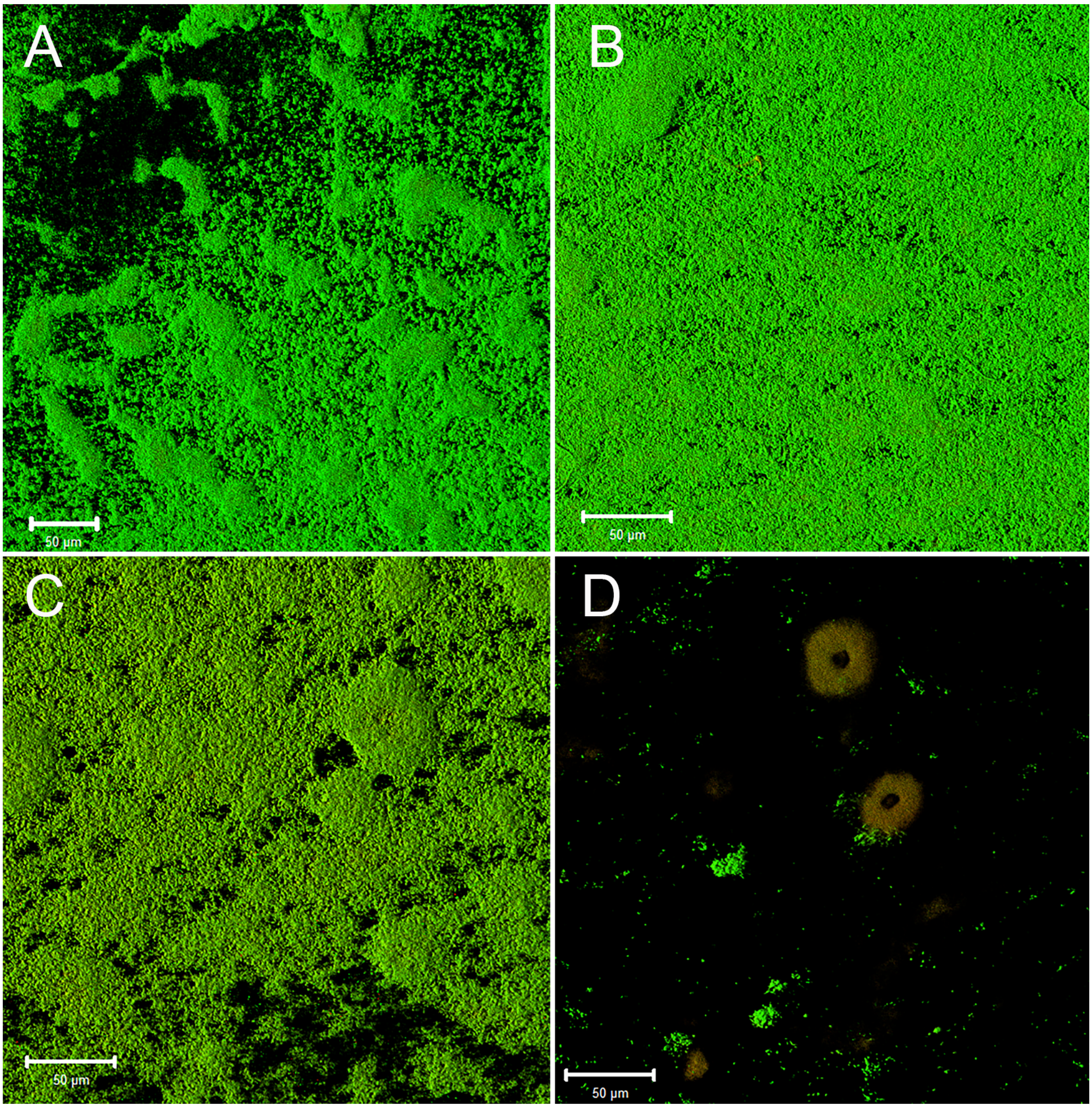


**Figure S5. Effect of colistin and N,N′-bis (2-hydroxybenzyl) ethylenediamine-N,N′-diacetic acid (HBED) on established *P. aeruginosa* PA605 biofilms.** *P. aeruginosa* PA605 was grown for 10 days with treatment (HBED (100 µM) and/or colistin (10 mg/L)) being incorporated into the media after 6 days. Biofilms were stained with LIVE/DEAD® stain and viewed using confocal scanning laser microscopy (CSLM). Scale bar = 50 µm. (a) Untreated (b) HBED (c) Colistin (d) HBED and colistin.
